# Diffuse Multibacillary Leprosy of Lucio and Latapí with Lucio’s Phenomenon, Peru

**DOI:** 10.3201/eid2311.171228

**Published:** 2017-11

**Authors:** Cesar Ramal, Martin Casapia, Johan Marin, Juan C. Celis, Jorge Baldeon, Stalin Vilcarromero, Guillermo Cubas, Alex Espejo, Francisco Bravo, Oswaldo V. Paredes, Jose M. Ramos, Pedro Legua

**Affiliations:** Loreto Regional Hospital, Iquitos, Peru (C. Ramal, M. Casapia, J. Marin, J.C. Celis, J. Baldeon, G. Cubas, A. Espejo, O.V. Paredes);; National University of the Peruvian Amazon, Iquitos (C. Ramal, M. Casapia, J. Baldeon);; U.S. Naval Medical Research Unit 6 (NAMRU-6), Iquitos (S. Vilcarromero);; Cayetano Heredia National Hospital, Lima, Peru (F. Bravo, P. Legua);; Alicante University General Hospital. Alicante, Spain (J.M. Ramos)

**Keywords:** Leprosy, multibacillary leprosy, lepromatous, diffuse leprosy of Lucio and Latapí, Lucio’s phenomenon, vascular thrombosis, Peru, Peruvian Amazon, South America, bacteria, *Mycobacterium* *leprae*

## Abstract

Diffuse multibacillary leprosy of Lucio and Latapí is mainly reported in Mexico and Central America. We report a case in a 65-year-old man in Peru. He also had Lucio’s phenomenon, characterized by vascular thrombosis and invasion of blood vessel walls by leprosy bacilli, causing extensive skin ulcers.

In Peru, leprosy has a prevalence of <1/10,000 inhabitants (*1*) and mainly affects people in the Peruvian Amazon (*2*). Leprosy, most commonly caused by infection with the bacterium *Mycobacterium leprae*, can be complicated by lepromatous reactions, which include the unusual manifestation known as Lucio’s phenomenon (*3*). Initially described by Lucio and Alvarado in 1852 in Mexico, Lucio’s phenomenon was so named in 1948 by Latapí and Zamoro (*3*). This reaction is seen in patients who have pure and primitive nonnodular lepromatous leprosy (diffuse leprosy of Lucio and Latapí [*4*]). This clinical variety of leprosy is most commonly found in Mexico and Central America (*5*–*7*) and is rarely described outside these regions (*8*–*10*). We report a case of multibacillary leprosy, specifically diffuse leprosy of Lucio and Latapí, and Lucio’s phenomenon in a patient from the Peruvian Amazon rainforest in Peru. 

The patient was a 65-year-old man who worked as a farmer. The disease had an insidious onset and a progressive course over several years, characterized by diffuse erythematous lesions on the skin. Leprosy was diagnosed, and the patient was prescribed treatment, but he did not take it. Two months before being admitted to a hospital, he began to experience a feeling of increased temperature and marked weakness. Multiple reddish-purple lesions with irregular borders appeared on his skin, mostly on the upper and lower extremities, and formed blisters and ulcers. Eventually, bacterial superinfection developed. The patient also experienced loss of appetite, gastric intolerance, and abdominal pain. Possible reasons the patient interrupted treatment were the stigmatization of the disease and a prohibitive distance from the patient’s residence to the health center, which complicated clinical supervision.

On physical examination after admission to the hospital, the patient was dehydrated, malnourished, and in poor general condition. There was diffuse infiltration of the skin on his face and ears, with partial loss of eyebrows and eyelashes, and diffuse erythematous infiltrative lesions on the chest. The upper and lower extremities had multiple reddish-purple lesions with irregular borders; multiple ulcers, some with purulent secretion and others with necrotic eschar; and some achromic scars (Figure, panel A). The patient’s fingers and toes were shortened and deformed (Figure, panel B) and his hands swollen. The ulcerated lesions were painful.

**Figure:**
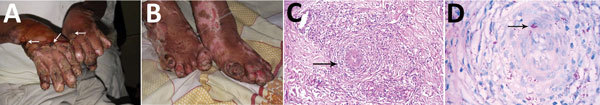
Diffuse multibacillary leprosy of Lucio and Latapí with Lucio’s phenomenon in a 65-year-old man in Peru. A) Vasculitis with necrosis of the superficial vascular plexus. Forearms and dorsum of hands show papulonodular dermotoses infiltrating erythematous lesions (white arrows). B) Patches of scaling skin or necrotic eschar on feet. C) Skin biopsy of leg (hematoxylin and eosin stain, original magnification ×40) showing largely unremarkable epidermis but collection of foamy histiocytes in dermis (arrow). D) Fite-Faraco staining (original magnification ×400) clearly shows large quantity of bacilli (arrows) in the foamy histiocytes, consistent with the theory of infectious vasculitis being an etiopathogenic mechanism of Lucio’s phenomenon.

We requested microscopic analysis (with an oil immersion objective lens at 1,000×) of earlobe skin samples. A minimum of 25 fields/sample were examined. These samples showed positive results (a value of +5, which was defined as 100–1,000 bacilli/observed field). The histopathological analysis, using punch biopsy of the knee, elbow, and shoulder, showed in all 3 fragments a moderate infiltrate of foam cells, which followed the linear paths of the blood vessels and nerves, in some areas that had occlusive vasculopathy and neutrophilic nuclear dust (Figure, panel C). We used Fite-Faraco staining (http://stainsfile.info/StainsFile/stain/micro/afb-fitefaraco.htm) to test for leprosy bacilli. Results were positive for *M*. *leprae* in the foam cells, interstitial cells, and endothelial cells (Figure, panel D). The anatomic pathology diagnosis was multibacillary leprosy with additional findings of Lucio’s phenomenon.

We prescribed the World Health Organization multidrug therapy (MDT) for multibacillary leprosy patients (http://www.who.int/lep/mdt/en/), comprising rifampin, clofazimine, and dapsone. The patient received treatment for 1 month in his home, and the dermal lesions improved considerably. He did not receive corticosteroid treatment. He was readmitted to the hospital with abdominal pain and severe gastric intolerance, which had forced him to discontinue MDT without consulting a doctor, probably within the previous month. The patient died from an undetermined cause.

Lucio’s phenomenon is defined as a variety of type 2 lepra reaction. It is a rare event that occurs in people with diffuse leprosy of Lucio and Latapí. It develops as a result of exacerbated proliferation of leprosy bacilli, which invade the walls of the blood vessels and the endothelial cells, causing endothelial proliferation and reduction of the vascular lumen. These effects, together with the inflammatory reaction and the changes in the coagulation system, lead to vascular thrombosis, ischemia, infarction, and necrosis of the tissues, giving rise to the histopathological features of the phenomenon (*4*,*6*).

The diagnosis of this patient was lepromatous leprosy, confirmed by a positive skin smear from the earlobes (5+). In addition, the patient’s condition met the 3 criteria that define Lucio’s phenomenon, according to the international literature: skin ulceration, vascular thrombosis, and invasion of blood vessels by leprosy bacilli (*4*,*6*,*9*). The term Lucio’s phenomenon should only be used when there is correlation of clinical and anatomic findings and in accordance with strict clinical criteria (*4*) The physiopathological mechanism of Lucio’s phenomenon requires further study to be properly understood (*7*). Lucio’s phenomenon is a serious condition that can worsen and result in patient death (*4*,*10*), as occurred with this patient.

Lucio’s phenomenon is a rare clinical form of multibacillary leprosy that may occur in a severe form in patients outside of Mexico and Central America. An effort must be made to look out for and treat the signs and symptoms as soon as possible to prevent an unfavorable course.
